# Strategies in activating lymphatic system on symptom distress and health-related quality of life in patients with heart failure: secondary analysis of a pilot randomized controlled trial

**DOI:** 10.3389/fcvm.2023.1248997

**Published:** 2023-09-19

**Authors:** Ruixia Liu, Jinbo Fang, Mei R. Fu, Qingtong Meng, Minlu Li, Xiaoxia Zhang, Sarah R. Allred, Yuan Li

**Affiliations:** ^1^ Department of Nursing, West China Hospital, Sichuan University/West China School of Nursing, Sichuan University, Chengdu, China; ^2^ School of Nursing and Health Studies, University of Missouri-Kansas City, Kansas City, MO, United States; ^3^ Department of Cardiology, Shenzhen People’s Hospital, Shenzhen, China; ^4^ General Ward of Neurology, West China Hospital, Sichuan University/West China School of Nursing, Sichuan University, Chengdu, China; ^5^ Division of Head & Neck Tumor Multimodality Treatment, Cancer Center, West China Hospital, Sichuan University/West China School of Nursing, Sichuan University/Innovation Center of Nursing Research, Nursing Key Laboratory of Sichuan Province, West China Hospital, Chengdu, China; ^6^ Department of Psychology and Health Sciences, The State University of New Jersey, Camden, NJ, United States; ^7^ Nursing Department, West China Second University Hospital, Sichuan University/West China School of Nursing, Sichuan University, Chengdu, China; ^8^ Key Laboratory of Birth Defects and Related Diseases of Women and Children (Sichuan University), Ministry of Education, Chengdu, China

**Keywords:** heart failure, lymphatic exercises, self-care, health related quality of life, symptom distress, randomized clinical trial

## Abstract

**Background:**

Abnormal interstitial fluid accumulation remains the major cause for patients with heart failure (HF) to endure a myriad of distressing symptoms and a decline in their health-related quality of life (HRQoL). The lymphatic system is essential in regulating fluid balance within the interstitial compartment and has recently been recognized as an important target for the prevention and mitigation of congestion. This study aimed to investigate the effects of exercises in activating lymphatic system on symptom distress and HRQoL among patients with HF.

**Methods and results:**

This was a pre-determined, secondary analysis of the *TOLF-HF* [*The-Optimal-Lymph-Flow for Heart Failure* (*TOLF-HF*)] study, a two-arm pilot randomized controlled trial evaluating the preliminary effects of the lymphatic exercise intervention in enhancing interstitial decongestion among patients with HF. Participants were randomized to receive either a four-week *TOLF-HF* program in addition to standard care or standard care alone. The Chinese version of the Minnesota Living with Heart Failure Questionnaire (MLHFQ) was employed to measure symptom distress and HRQoL before and after the intervention. Data analyses included descriptive statistics, the independent sample *t*-test, Pearson’s chi-square test, the Mann-Whitney *U* test, and covariance analysis. Of the 66 patients enrolled, 60 completed the study. The study results exhibited that the *TOLF-HF* intervention were effective in alleviating both physical and psychological symptom distress. The intervention group yielded significantly lower MLHFQ total scores in comparison to the control group. The odd ratio of achieving meaningful improvement in HRQoL in *TOLF-HF* group was 2.157 times higher than those in the control group.

**Conclusions:**

The *TOLF-HF* program focusing on activating lymphatic system was effective in alleviating physical and psychological symptom distress as well as improving HRQoL for patients with HF. The tolerability, feasibility, and effectiveness of the *TOLF-HF* intervention make it a promising intervention for patients to manage HF.

**Clinical Trial Registration:**

http://www.chictr.org.cn/index.aspx, identifier (ChiCTR2000039121).

## 1. Introduction

Heart failure (HF) is a significant public health challenge, affecting an estimated 64 million individuals worldwide (1). The prevalence of HF is age-dependent, ranging from under 2% among people younger than 60 years to exceeding 10% among those aged 80 years or older (2). With the rapidly aging global population, the prevalence of HF continues to increase worldwide. Abnormal interstitial fluid accumulation is central to the pathophysiology of HF, leading to hemodynamic congestion characterized by elevated central filling pressures and the subsequent onset of clinical congestion (3). Clinical congestion manifests as a myriad of congestive symptoms that elicit distress to the patient (4, 5). The occurrence or exacerbation of congestive symptoms constitutes a major contributor to diminished health-related quality of life (HRQoL) (6). According to time trade-off utility studies, patients suffering from HF would be willing to exchange their lifespan for an enhanced quality of life, with individuals experiencing severer symptoms being more inclined to prioritize HRQoL over longevity (7, 8). To help manage symptom distress and improve HRQoL in patients suffering from HF, it is imperative to provide feasible and effective interventions to prevent and mitigate congestion.

The lymphatic system, an integral part of the circulatory system, plays a crucial role in maintaining the balance of tissue fluid levels by taking up interstitial fluid in the form of lymph and returning it into the central circulation (9, 10). However, in the context of HF, the elevated central venous pressure leads to increased fluid accumulation in the interstitial space, and simultaneously impedes fluid to flow back into the venous system (9). Moreover, a reduction in the quantity of lymphatic vessels, accompanied by an expansion of their diameters in HF, also contributes to the accumulation of fluid within the interstitial space (11). Congestion occurs when the drainage of fluid fails to match the rate at which it permeates into the interstitial spaces (12). Therefore, the activation of the lymphatic system to facilitate lymphatic circulation with enhanced permeation holds great potential for averting and alleviating subclinical and clinical congestion. This potential is rooted in the fact that the removal of interstitial fluid relies exclusively on lymphatic pumping, and the lymphatic system has the capacity to augment fluid elimination by a minimum of ten-fold (9, 10, 13).

*The-Optimal-Lymph-Flow* (*TOLF*) intervention comprises a set of non-pharmacological therapeutic exercises that specifically target the activation of the lymphatic system (14–18). The fundamental *TOLF* intervention comprises lymphatic exercises which entail the contraction of muscles and pumping movements that are synchronized with deep breathing to simulate the physiological process of lymphatic pumping (14–18). *TOLF* lymphatic exercises are designed to elicit musculoskeletal contractions, skin tensions, arterial pulsations, postural adjustments, and modifications in breathing patterns; all of these collectively serve to activate the lymphatic system (14–18). As a result, the *TOLF* intervention possesses great promise for yielding favorable outcomes in the prevention and management of congestion.

*The-Optimal-Lymph-Flow for Heart Failure* (*TOLF-HF*) trial was conducted to assess the preliminary effects of the four-week lymphatic exercise training program among patients afflicted with HF. The finding revealed that the innovative implementation of *TOLF-HF* program was beneficial in alleviating the burden of congestive symptoms, enhancing physical functions, and maintaining stable body weight for this population (16). Nevertheless, it is unknown whether this strategy have positive impact on symptom distress and HRQoL, which were predefined as secondary outcomes in the *TOLF-HF* trial. In this analysis, we evaluated the effects of *TOLF-HF* intervention compared to standard care on the overall summary score and symptom distress items of the Minnesota Living with Heart Failure Questionnaire (MLHFQ).

## 2. Materials and methods

### 2.1. Study design and population

**Figure 1:**
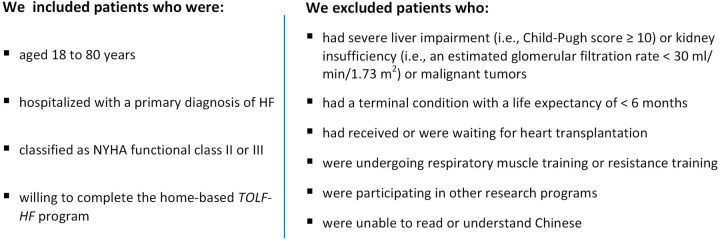
The inclusion and exclusion criteria.

The detailed information regarding the design of the *TOLF-HF* trial was previously published (16). In brief, the *TOLF-HF* trial was a single-center, two-arm, parallel-group, pilot randomized controlled trial (RCT) in which eligible patients were between the ages of 18 and 80, admitted to the hospital with a primary diagnosis of HF. The diagnosis of HF was made based on the Chinese guidelines for the diagnosis and treatment of heart failure 2018 (19). The detailed inclusion and exclusion criteria are given in Figure 1. Consecutive identification of eligible participants was carried out in the West China Hospital by reviewing inpatient census lists. Prospective participants were presented with the study details two days prior to their discharge. Patients who signed the informed consent form to participate in the study completed the baseline assessment.

The study randomly assigned participants in a 1:1 ratio to receive either standard care or a four-week *TOLF-HF* program in addition to standard care. The randomization assignment was determined using the SPSS random number generator by an independent researcher. Allocation concealment was assured through the use of sequentially numbered, sealed, opaque envelopes containing group assignment. Blinding of participants and interventionists was impossible given the inherent nature of the intervention in the study. Nevertheless, the outcome assessor, data collector, and data analyst were blinded to the group assignment throughout the entire duration of the study.

### 2.2. Interventions

Participants allocated to the control group received standard care. Apart from the implementation of guideline-directed medical therapy for managing HF (19, 20), the standard care included providing patients with a comprehensive written summary containing essential information about their medical condition and treatment plan upon discharge. Moreover, nurses provided verbal guidance on adopting healthy lifestyle behaviors and adhering to prescribed medications. If it is deemed necessary, patients can be directed to post-discharge support services. Following their discharge, all participants were scheduled to attend a specialist clinic for a follow-up visit after four weeks. No additional educational or supportive post-discharge care was administered.

**Figure 2:**
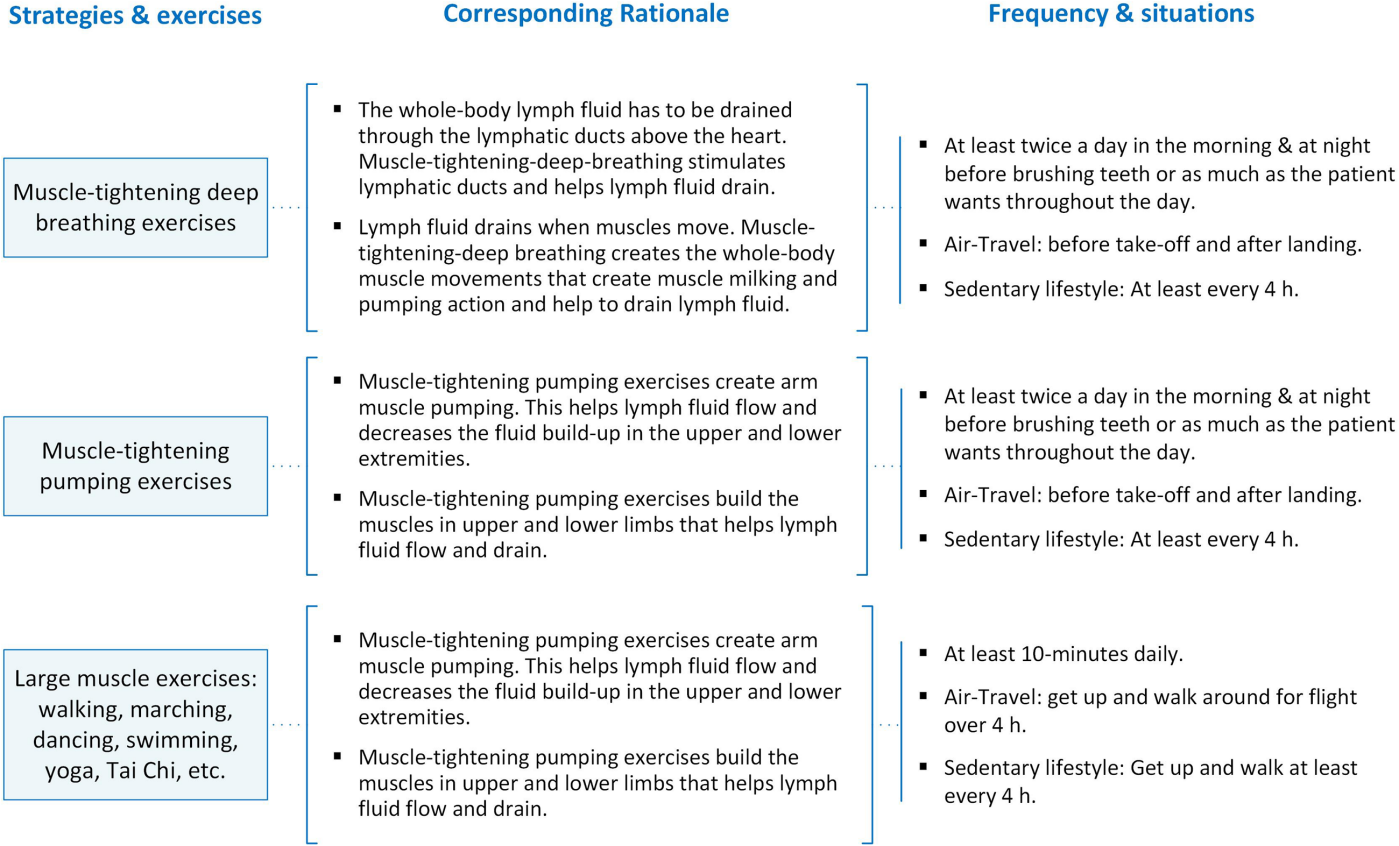
*TOLF-HF* program strategies and rationales.

Participants assigned to the experimental group underwent the four-week *TOLF-HF* intervention plus standard care. The *TOLF-HF* program was fashioned based on physiological-cognitive-behavioral principles, featuring the activation of the lymphatic system through effective self-care strategies to ameliorate the pathological status of congestion in patients suffering from HF (14–18). Figure 2 provides an overview of the strategies employed in the TOLF-HF program, along with their corresponding physiological rationales and the recommended frequency of practice. Specifically, muscle-tightening deep breathing exercises serve to activate lymphatic ducts, thereby facilitating the drainage of lymph fluid. Muscle-tightening pumping exercises aid in promoting the flow of lymph fluid and mitigating fluid retention in the extremities. Furthermore, engaging in large muscle exercises enhances the flow and drainage of lymph fluid throughout the entire body (14–18).

The implementation of the *TOLF-HF* program was promptly initiated following the baseline assessment and conducted within two days prior to hospital discharge. The intervention was administered by a trained researcher during a dedicated 30-minute one-to-one session. Initially, a detailed video presentation was utilized to provide clear and standardized step-by-step instructions on the correct execution and frequency of the lymphatic exercises. Subsequently, patients were required to practice independently while being closely observed by the researcher, who promptly corrected any improper movements until patients were able to perform all exercises accurately. During the patients’ performance of lymphatic exercises, the researcher recorded a video clip, which was then shared with the patients for future reference as needed. The involvement of family members was encouraged throughout the process, with their active participation in accompanying and supporting the patients’ practice at home. Following hospital discharge, the researcher maintained weekly contact via WeChat to address any potential barriers, provide relevant advice, and encourage adherence to the *TOLF-HF* protocol.

### 2.3. Measurements

#### 2.3.1. Participant characteristics

A structured questionnaire was used to collect participants’ sociodemographic and clinical information. Sociodemographic information included age, gender, educational attainment, occupational status, and caregiver role. Clinical information included weight, height, body mass index, blood pressure, heart rate, length of hospital stays, duration of HF, HF etiology, HF type, number of co-morbidities, left ventricular ejection fraction, New York Heart Association function class, NT-proBNP levels, and dose of diuretics.

#### 2.3.2. Health-related quality of life and symptom distress

HRQoL was measured with the Minnesota Living with Heart Failure Questionnaire (MLHFQ), which is widely utilized as a disease-specific instrument that captures the impact of HF on patients’ daily life and functioning (21). This 21-item questionnaire, assessed on a 6-piont Likert scale of 0 (“no impact”) to 5 (“very high impact”), provides a summary score that ranges from 0 to 105, with higher scores indicating a lower HRQoL. Five points is generally considered as the minimal clinically important difference in overall MLHFQ score (22). The Chinese version of the MLHFQ has undergone validation and exhibited satisfactory reliability, with a Cronbach’s alpha coefficient of 0.95 (23).

Eight items within the MLHFQ were specifically designed to assess the degree of distress associated with HF symptoms, that is, to which extent these symptoms have prevented individuals from living as they wanted (24, 25). In the current analysis, these items were utilized to measure the patients’ symptom distress together with the evaluation of their overall HRQoL. Among these eight items, five specifically address physical symptoms (i.e., swelling in the ankles or legs, resting during days, sleeping difficult, shortness of breath, fatigue), while the remaining three focus on psychological symptoms (i.e., being worried, difficulty concentrating or remembering, being depressed) (24, 25). The scores for these items were derived from the overall MLHFQ assessment, where higher scores correspond to elevated levels of distress attributed to the symptoms (23).

### 2.4. Data collection

Patient self-report and electronic medical records were utilized to gather baseline sociodemographic and clinical data. The outcome measures were completed by all participants both at baseline and during their routine visits to the specialist clinic, which occurred 4 weeks after discharge. An impartial research assistant conducted data collection without knowledge of the study’s hypothesis or participant allocation.

### 2.5. Statistical analysis

Descriptive analyses were conducted to present the baseline characteristics of the participants, stratified by treatment group. The Shapiro-Wilk test was employed to assess the normality of the data, while the Levene test was used to evaluate the homogeneity of variance. Continuous variables were reported as means with standard deviations (SDs) or medians with interquartile ranges (IQRs), depending on the distribution of the data. The comparison of the intervention group versus control group was conducted using the independent sample *t*-test for variables that exhibited a normal distribution. In cases where the variables did not adhere to normal distribution, the Mann-Whitney *U* test was employed instead. Where appropriate, covariance analysis (ANCOVA) was also performed to account for baseline outcome measures and significant demographic covariates. Categorical variables were reported as counts with percentages and analyzed using Pearson’s chi-square test. All statistical analyses were conducted using SPSS for Windows 26.0. Statistical significance was defined as a two-sided *p* value of <0.05.

### 2.6. Ethical considerations

The study was approved by the Biomedical Ethics Committee of the West China Hospital, Sichuan University [Approval number: 2019 (202)]. This study was registered with the Chinese Clinical Trial Registry (registration number ChiCTR2000039121) and adhered to the reporting guidelines outlined in the CONSORT statement (26). Detailed information of the study was provided with each potential participant, including the objectives, methods, anticipated duration, potential hazards, advantages, and the option to decline or withdraw from participation. Ample time was provided for potential participants to read the informed consent and ask questions. Participants were also assured that their personal data would remain confidential and not be disclosed to any third parties. Written informed consent was obtained from all participants.

## 3. Results

Participant enrollment for this pilot study spanned from March 2019 to January 2020. The CONSORT flow diagram can be found in the first publication from the *TOLF-HF* study (16) and is provided in Supplementary Figure S1. Of the 85 patients assessed for eligibility, 66 met the inclusion criteria and were enrolled in the trial. During the study period, 6 patients were lost to follow-up. The final analysis was conducted using data from 60 patients (29 in *TOLF-HF* group and 31 in control group) who provided complete information.

### 3.1. Participant characteristics

The mean age was 58.07 years old [standard deviation (SD) = 12.79] for the *TOLF-HF* group and 61.65 years old (SD = 11.42) for the control group. Within each group, 65.5% and 64.5% were male, 75.9% and 64.5 were diagnosed as HF with reduced ejection fraction (HFrEF), and 37.9% and 58.1% of the participants were classified as NYHA class III, respectively. Detailed information on study participant characteristics is described in Supplementary Table S1 (16). No statistically significant differences in demographic and clinical characteristics were found between intervention and control groups at baseline, except that patients in intervention group had a higher prevalence of co-morbidities versus the control group (*Z* = 2.449, *p* = 0.014).

### 3.2. Effects of the intervention on symptom distress

As indicated in Table 1, no statistically significant between-group differences were found at baseline in terms of median distress scores for each physical and psychological symptom. At four weeks post-intervention, the *TOLF-HF* group exhibited significantly lower median physical symptom distress scores compared to the standard care control group for swelling in the ankles or legs (*p* = 0.036), sleeping difficult (*p* = 0.041), and shortness of breath (*p* = 0.003). While no significant differences were noted in symptom distress for resting during days (*p* = 0.108) and fatigue (*p* = 0.113). Regarding psychological symptoms, the *TOLF-HF* group demonstrated significantly lower median psychological symptom distress scores for being worried (*p* = 0.002) and difficulty concentrating or remembering (*p* = 0.016) after the intervention. However, no significant differences were seen for being depressed (*p* = 0.292).

**Table 1:** Comparison of between-group differences in symptom distress (*n* = 60).

Variables			*TOLF-HF* group (*n* = 29), median (IQR)	Standard care group (*n* = 31), median (IQR)	*Z*	*p* *
Physical symptoms	Swelling in the ankles or legs	Baseline	1.00 (0.00–4.00)	2.00 (0.00–4.00)	−0.883	0.337
		Endpoint	0.00 (0.00–1.00)	1.00 (1.00–2.00)	−2.046	0.036
	Resting during days	Baseline	3.00 (1.00–4.00)	3.00 (1.00–4.00)	−0.068	0.946
		Endpoint	0.00 (0.00–1.00)	1.00 (0.00–3.00)	−1.607	0.108
	Sleeping difficult	Baseline	4.00 (3.00–5.00)	2.00 (0.00–4.00)	−1.805	0.071
		Endpoint	1.00 (0.00–1.00)	3.00 (0.50–3.50)	−2.044	0.041
	Shortness of breath	Baseline	3.00 (0.00–4.00)	4.00 (2.00–5.00)	−1.862	0.063
		Endpoint	1.00 (1.00–2.00)	2.00 (1.00–4.00)	−3.031	0.003
	Fatigue	Baseline	3.00 (1.00–4.00)	3.00 (2.00–4.00)	−1.023	0.306
		Endpoint	2.00 (1.00–3.00)	3.00 (2.00–3.00)	−1.584	0.113
Psychological symptoms	Being worried	Baseline	1.00 (0.00–3.00)	2.00 (1.00–3.00)	−0.833	0.405
		Endpoint	0.00 (0.00–1.00)	2.00 (0.00–3.00)	−3.028	0.002
	Difficulty concentrating or remembering	Baseline	1.00 (0.00–3.00)	1.00 (0.00–2.50)	−0.146	0.884
		Endpoint	0.00 (0.00–1.00)	1.00 (0.00–2.50)	−2.048	0.016
	Being depressed	Baseline	1.00 (0.00–3.00)	1.00 (0.00–3.00)	−0.031	0.976
		Endpoint	1.00 (0.00–1.00)	1.00 (0.00–2.00)	−1.053	0.292

* *p*-values were generated using the Mann-Whitney *U* test.

### 3.3. Effects of the intervention on health-related quality of life

**Figure 3:**
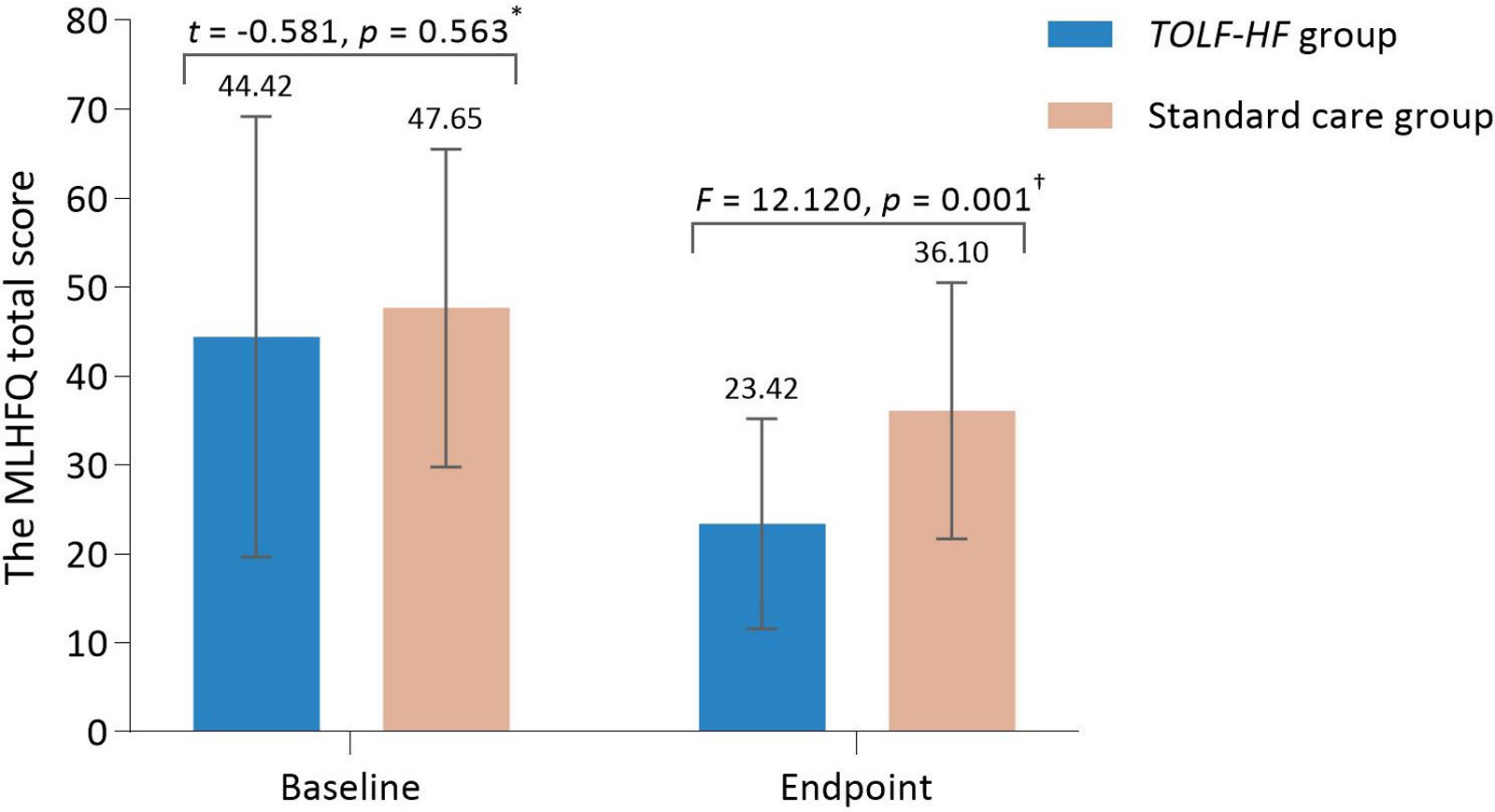
Comparison of between-group differences in health-related quality of life (*n* = 60). *The data were compared using the independent sample *t*-test.^†^The data were compared using the analysis of covariance (ANCOVA) after controlling for the pre-intervention health-related quality of life values and the number of co-morbidities.

**Figure 4:**
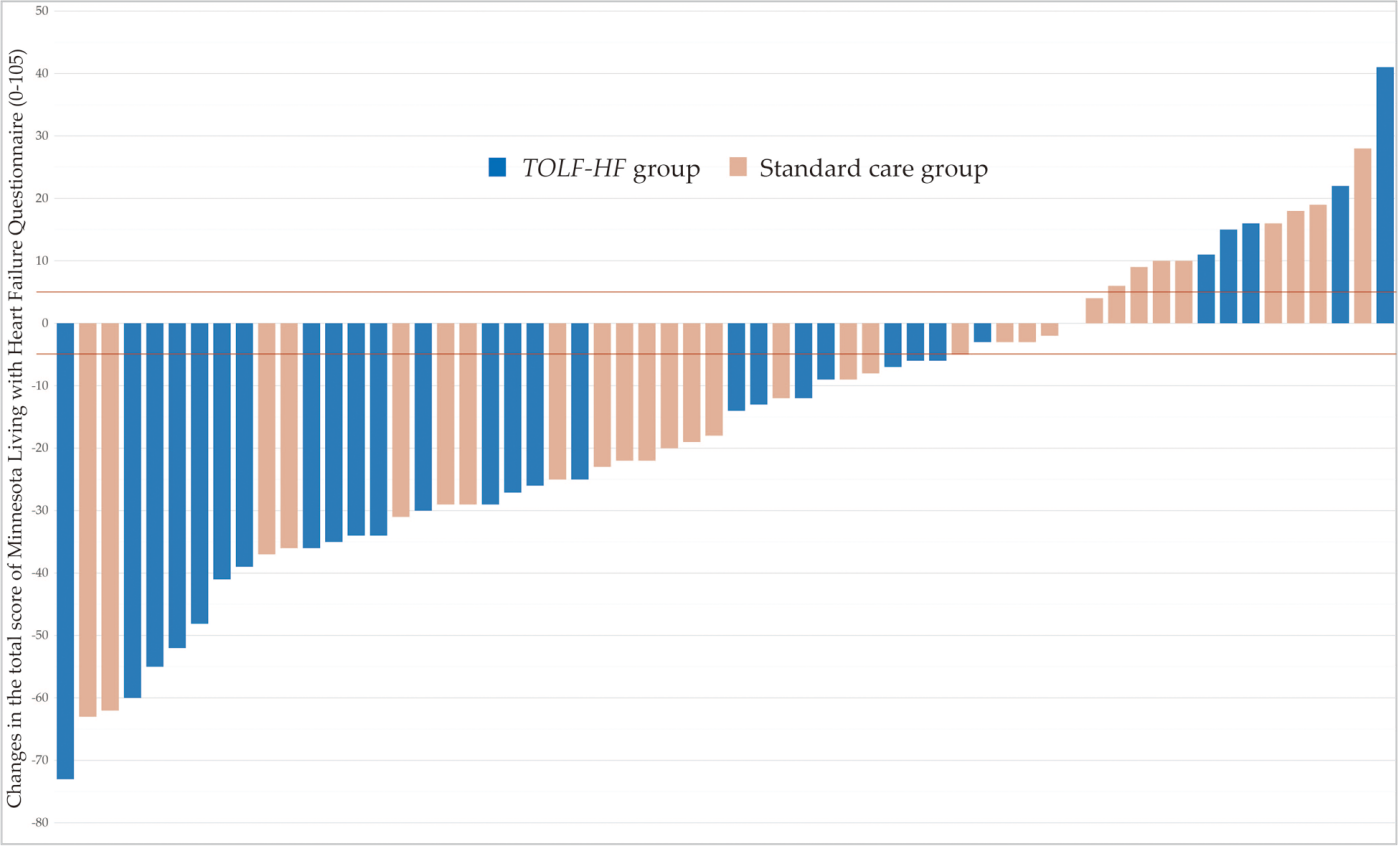
Inter-individual variation in the change of MLHFQ total score for each patient of the *TOLF-HF* and control groups (*n* = 60). The lines represent the minimal clinically important difference for improvement (−5 points) and worsening (+5 points) of health-related quality of life in patients with heart failure.

As illustrated in Figure 3, no significant differences were detected between the groups with regards to the mean MLHFQ total scores at baseline. At the study endpoint, the MLHFQ total scores were significantly lower in the *TOLF-HF* group compared to the standard care control group (*F *= 12.120, *p* = 0.001), indicating an improved HRQoL for patients who underwent the intervention. Additionally, the inter-individual variation in the change of MLHFQ total score from baseline to week 4 is shown as a waterfall plot in Figure 4. It was noted that 79.3% (23/29) of the patients in the *TOLF-HF* group achieved a clinically important improvement, compared to 54.8% (17/31) in the standard care group. The odds ratio of attaining a meaningful improvement in HRQoL in the *TOLF-HF* group was 2.157 times (95% CI 1.006–9.906; *p* = 0.044) higher than that in the standard care group.

## 4. Discussion

The challenge in visualizing the translucent lymphatic vessels has been historically led to overlook the significant role of the lymphatic system in regulating fluid homeostasis. Recent research progressions on the lymphatic network and the pathogenesis of cardiovascular diseases have established the importance of targeting the lymphatic circulation in the management and treatment of cardiovascular disorders (27, 28). The *TOLF-HF* trial innovatively applied lymphatic exercises among patients with HF. In this pre-determined secondary analysis of the pilot trial, we observed that patients who underwent *TOLF-HF* intervention demonstrated a reduction in physical and psychological symptom distress, as well as an improvement in HRQoL. While the original paper focused on the frequency, severity, and burden of congestion-related symptoms, this secondary analysis provides a more comprehensive look at the distress associated with overall HF symptoms. The findings were complementary to the primary analysis (16) and provided further support that the *TOLF-HF* program focusing on promoting lymph fluid flow through therapeutic lymphatic exercises is an effective adjunctive therapy for individuals with HF in terms of managing symptom distress and improving HRQoL.

### 4.1. The *TOLF-HF* program was effective in improving symptom distress

Patients with HF commonly endure a multitude of distressing physical symptoms attributed to congestion (24, 29). The study findings showed that the integration of the four-week *TOLF-HF* lymphatic exercises, in conjunction with standard care, had beneficial effects on mitigating the levels of symptom distress associated with lower extremity swelling, sleep difficulty, and shortness of breath in individuals with HF. The amelioration of the aforementioned symptom distress can be attributed to the activation of lymphatic system and the consequent reduction in extracellular fluid retention. Specifically, the relief of distress related to lower extremity swelling can be ascribed to the enhanced clearance of fluid accumulation in the systemic circulation, while the alleviation of sleep difficulty and shortness of breath can be attributed to the improved resolution of volume overload in the pulmonary circulation (30). The *TOLF-HF* program, comprising muscle tightening deep breathing, muscle tightening pumping, and large muscle exercises developed to emulate the physiological mechanism of lymph propulsion (14–18), creates a synergistic effect in accelerating the removal of fluid volume in both the thoracic region and the whole body. Thus, the intervention confers benefits to patients by ameliorating physical symptoms associated with congestion.

Physical and psychological symptoms often co-exist in HF (24, 29). The study demonstrated that the *TOLF-HF* program yielded favorable outcomes in mitigating the levels of psychological symptom distress associated with being worried and difficulty concentrating or remembering. These psychological symptoms serve as indicators of the anxiety status in individuals with HF. The *TOLF-HF* lymphatic exercises encompass tightening and pumping movements that are coordinated with deep breathing. A previous RCT conducted by D’Silva et al. found that deep breathing exercises was beneficial in decreasing anxiety in patients with coronary heart disease (31). Another RCT also demonstrated the positive impact of deep breathing exercises on anxiety levels in patients with gestational diabetes (32). Furthermore, deep breathing constitutes an essential component of mindfulness-based interventions, which have consistently demonstrated effectiveness in alleviating anxiety across diverse populations (33–36). Accordingly, the relief of being worried and difficulty concentrating or remembering in the current study can be explained by the positive effect of deep breathing on psychological status. Furthermore, the beneficial effects of the *TOLF-HF* intervention on physical symptoms could potentially function as a mediator in alleviating psychological distress.

### 4.2. The *TOLF-HF* program was effective in improving health-related quality of life

HRQoL is an important outcome that reflects the impact of HF on patients’ daily life (21). The study results indicated that the *TOLF-HF* intervention have positive effects on the overall HRQoL for patients suffering from HF. The likelihood of achieving clinically meaningful improvement from baseline in overall HRQoL was two times greater in patients who received the intervention than those received standard care alone. The finding is consistent with a previous Cochrane review, which provided a comprehensive review of exercise-based intervention for adults with HF and showed positive effects on HRQoL (37). The positive effects can be attributed to the beneficial impact of *TOLF-HF* exercises in ameliorating prevalent physical symptoms, which are widely recognized as independent predictors of HRQoL (38, 39). *TOLF-HF* exercises also provide psychological benefits among the participants and emotional well-being serves as an important aspect of HRQoL (39). Moreover, it is noteworthy that the intervention encourages these participating patients to pursue an active lifestyle, which is widely regarded as one of the most beneficial measures individuals can take to prevent illness, maintain good health, and improve their HRQoL (40, 41). Additionally, engaging in exercise-based interventions empowers HF patients to take a proactive role in managing their condition. As they experience improvements in symptom management, physical functioning, and psychological well-being, patients may gain a sense of control over their health, resulting in enhanced HRQoL (42, 43).

### 4.3. The advantages of the *TOLF-HF* program

The *TOLF-HF* program exhibits multiple advantages. Firstly, the intervention is easy to learn and does not require the use of costly equipment or constant supervision of healthcare providers. Moreover, the *TOLF* lymphatic exercises can be performed in any desired location, granting individuals the flexibility to engage in the exercises according to their preferences. With proper training, virtually anyone can learn and perform *TOLF* lymphatic exercises in a preferred setting (e.g., indoor or outdoor) or preferred positions (e.g., sitting, standing, or lie-down position). This makes the intervention particularly suitable for the HF population, as a significant number of HF patients are elderly and may have physical and cognitive impairments which limit their ability to perform 30 min walking or exercises that need standing position. Additionally, the *TOLF-HF* lymphatic exercises are of relatively low intensity and do not impose high physical demands. Thus, even patients who are immobilized or bedridden can perform the majority of these exercises. The tolerability, feasibility, and effectiveness of the *TOLF-HF* intervention make it a promising candidate for integration into daily self-care regimens for HF management.

In addition, it is worth noting that the management of extravascular volume overload is progressively gaining recognition as a therapeutic target in HF (13). New device-based treatments are currently under development to restore interstitial fluid balance and alleviate congestive symptoms in HF patients (44). For example, the alfapump DS® (Sequana Medical NV, Belgium) (45) and the Reprieve System™ (Reprieve Cardiovascular, Milford, MA, USA) (46) exemplify such emerging technologies, which have demonstrated safety and tolerability in preliminary exploratory clinical trials. As we anticipate the translation of these innovative device-based treatments into clinical practice, it is imperative to underscore the advantages inherent in the *TOLF-HF* program. These advantages encompass enhanced convenience, in-home accessibility, cost-effectiveness, and preventive orientation compared to device-based volume removal treatments that necessitate invasive procedures and professional oversight.

### 4.4. Study limitations

We acknowledge the limitations of our study, including its single-center design, short follow-up duration, and small sample size. It is important to note that the beneficial effects of *TOLF-HF* were found in patients in the intervention group even though a higher proportion of individuals within the intervention group presented with a greater number of co-morbidities at the baseline and we also accounted for this using analysis of covariance. Another limitation of the study is the absence of real-time monitoring of the actual exercise dose administered, which hinders the trial’s ability to investigate the dose-effectiveness of the intervention. Further validation of the study findings is warranted through larger multicenter studies with a larger sample size, longer follow-up periods, and comprehensive exercise monitoring. Moreover, future studies may benefit from the incorporation of more objective metrics, such as echocardiography and chest x-ray, to directly quantify the decongestion impact of the *TOLF-HF* intervention.

## 5. Conclusions

The *TOLF-HF* program has demonstrated effectiveness in reducing both physical and psychological symptom distress and improving HRQoL in patients with HF, and shows promise as a candidate for integration into daily self-care regimens for HF management. The *TOLF-HF* trial represents an initial and important endeavor in targeting the lymphatic system as a potential approach to managing congestion in HF. Moving forward, further research involving larger samples is warranted to verify the benefits of *TOLF-HF* and explore its long-term clinical impacts. Nevertheless, this pilot trial has provided valuable preliminary evidence and laid a solid foundation for utilizing lymphatic activation as a new self-care modality for patients with HF.

## Data Availability

The raw data supporting the conclusions of this article will be made available by the authors, without undue reservation.
