# Supplementary material for: Strategies in activating lymphatic system on symptom distress and health-related quality of life in patients with heart failure: secondary analysis of a pilot randomized controlled trial
Source: Front Cardiovasc Med. 2023 Sep 19;10:1248997. doi: 10.3389/fcvm.2023.1248997 (PMC10546325; doi:10.3389/fcvm.2023.1248997)
Supplement: Supplementary file 1 [file Datasheet1.docx]

Supplementary Material

*** Correspondence:** Yuan Li: li.yuan@scu.edu.cn

# Supplementary Figures and Tables

## Supplementary Figures


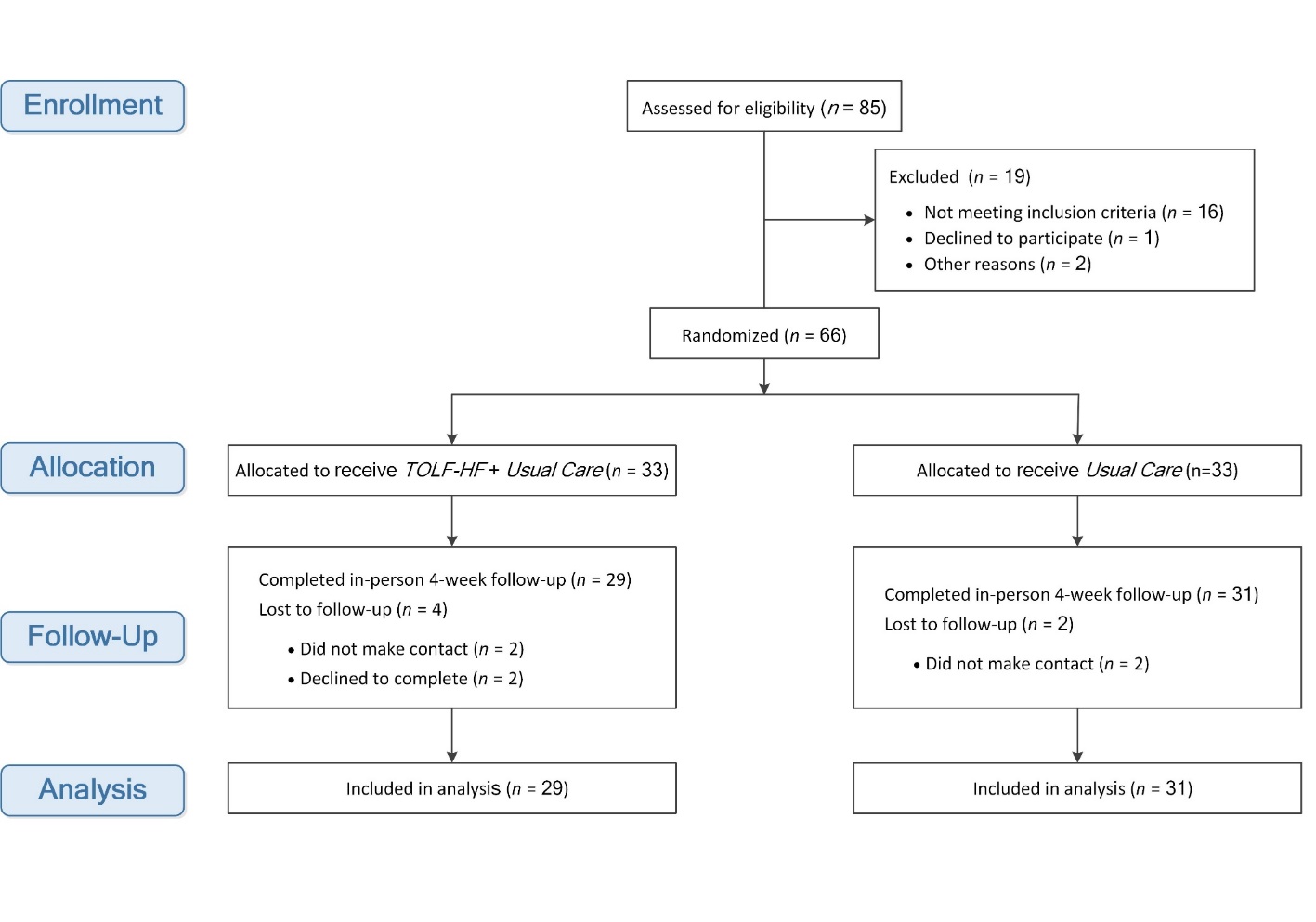


**Supplementary Figure 1.** The CONSORT flow diagram.

## Supplementary Tables

**Supplementary Table 1.** Sociodemographic and clinical characteristics of participants at baseline (n = 60).

| Variables | *TOLF-HF* group  (n = 29) | Standard care group  (n = 31) | *t*/χ^2^/*Z* | *df* | *p* |
| --- | --- | --- | --- | --- | --- |
| Age, mean (SD), years | 58.07 (12.79) | 61.65 (11.42) | −1.144 | 58 | 0.257 |
| Gender, n (%) |  |  | 0.000 | 1 | 1.000 |
| Male | 19 (65.5) | 20 (64.5) |  |  |  |
| Female | 10 (34.5) | 11 (35.5) |  |  |  |
| Level of education, n (%) ^†^ |  |  | 1.854 | 3 | 0.603 |
| No formal education | 4 (13.8) | 2 (6.5) |  |  |  |
| Elementary school | 6 (20.7) | 8 (25.8) |  |  |  |
| Secondary school | 10 (34.5) | 8 (25.8) |  |  |  |
| College/university and above | 9 (31.0) | 13 (41.9) |  |  |  |
| Occupational status, n (%) |  |  | 0.071 | 1 | 0.789 |
| Employed | 16 (55.2) | 15 (48.4) |  |  |  |
| Unemployed | 13 (44.8) | 16 (51.6) |  |  |  |
| Caregiver, n (%) |  |  | 4.447 | 3 | 0.206 |
| Self (no caregiver) | 0 (0) | 2 (6.5) |  |  |  |
| Spouse/partner | 5 (17.2) | 1 (3.2) |  |  |  |
| Offspring | 12 (41.4) | 15 (48.4) |  |  |  |
| Paid caregiver | 12 (41.4) | 13 (41.9) |  |  |  |
| Weight, mean (SD), kg | 60.27 (12.24) | 58.97 (15.80) | 0.347 | 56 | 0.730 |
| Height, mean (SD), cm | 165.11 (8.35) | 164.78 (8.42) | 0.153 | 56 | 0.879 |
| BMI, mean (SD), km/m^2^ | 22.04 (3.76) | 21.72 (5.27) | 0.266 | 56 | 0.791 |
| SBP, mean (SD), mmHg | 110.76 (20.85) | 114.07 (17.57) | −0.666 | 58 | 0.508 |
| DBP, mean (SD), mmHg | 75.90 (16.07) | 76.19 (12.93) | −0.079 | 58 | 0.937 |
| HR, mean (SD), bpm | 91.00 (21.49) | 89.35 (17.40) | 0.327 | 58 | 0.745 |
| Length of hospital stay, mean (SD), days | 10.28 (4.20) | 9.52 (2.55) | 0.853 | 58 | 0.397 |
| Duration of HF, median (IQR), years | 2.00 (0.00-6.00) | 1.00 (0.00-4.00) | 0.169 |  | 0.865 |
| HF etiology, n (%) |  |  | 1.473 | 4 | 0.914 |
| Cardiomyopathy | 15 (51.7) | 20 (64.5) |  |  |  |
| Valvular heart disease | 6 (20.7) | 5 (16.1) |  |  |  |
| Hypertension | 4 (13.8) | 3 (9.7) |  |  |  |
| ischemic heart disease | 3 (10.3) | 2 (6.5) |  |  |  |
| Others | 1 (3.4) | 1 (3.2) |  |  |  |
| Number of co-morbidities, median (IQR) | 2.0 (1.0-3.0) | 1.0 (0.0-2.0) | 2.449 |  | 0.014 |
| LVEF, median (IQR), % | 27.0 (22.5-38.5) | 35.0 (23.0-44.0) | –1.066 |  | 0.286 |
| HF type, n (%) |  |  | 1.630 | 2 | 0.433 |
| HFrEF | 22 (75.9) | 20 (64.5) |  |  |  |
| HFmrEF | 3 (10.3) | 7 (22.6) |  |  |  |
| HFpEF | 4 (13.8) | 4 (12.9) |  |  |  |
| NYHA function class, n (%) |  |  | 1.693 | 1 | 0.193 |
| Grade II | 18 (62.1) | 13 (41.9) |  |  |  |
| Grade III | 11 (37.9) | 18 (58.1) |  |  |  |
| NT-proBNP, median (IQR), pg/ml | 1,626.0 (673.0-8,127.0) | 3,075.0 (1,389.5-4,863.5) | −1.123 |  | 0.261 |
| Dose of diuretics, medi*a*n (IQR), mg/day | 80.00 (40.0–120.0) | 40.00 (40.0–80.0) | 1.341 |  | 0.180 |

Abbreviations: *df*, degree of freedom; SD, standard deviation; BMI, body mass index; SBP, systolic blood pressure; DBP, diastolic blood pressure; HR, heart rate; IQR, inter-quartile range; LVEF, left ventricular ejection fraction; HFrEF, heart failure with reduced ejection fraction; HFmrEF, heart failure with mid-range ejection fraction; HFpEF, heart failure with preserved ejection fraction; NYHA, New York Heart Association; NT-proBNP, N-terminal pro-brain natriuretic peptide.
